# Risk Assessment of Haier Group’s Overseas Investment Under International Financial Reporting Standards

**DOI:** 10.3389/fpsyg.2022.889670

**Published:** 2022-05-20

**Authors:** Bin Zhong, Wei Ni Soh, Tze San Ong, Haslinah Bt. Muhammad, Chun Xi He

**Affiliations:** School of Business and Economics, Universiti Putra Malaysia, Serdang, Malaysia

**Keywords:** overseas investment, international financial reporting standards, risk assessment, risk analysis model, market effect

## Abstract

With the development of economic globalization and the policy guidance of International Financial Reporting Standards (IFRS), the overseas investment of Chinese enterprises has been greatly affected. To study the overseas investment risks of Chinese enterprises, this paper applies a risk analysis model to summarize and analyze the results of overseas investment of *Haier* from 2008 to 2020. This paper defines the risk analysis model as risk identification, risk assessment, and risk response, and studies overseas investment risks including political, economic, cultural, legal, cost, epidemic, and business risks. (1) The existence of various risks is identified in the company’s overseas investment projects; (2). The overseas investment risks of *Haier* are evaluated, through a comprehensive evaluation method combining qualitative and quantitative, financial data, and market response; (3). In view of the deficiencies of the existing risk prevention measures in the overseas investment of enterprises, some reasonable suggestions are purposed. The innovation of this paper are: This paper combines IFRS 9, IFRS 15, and IFRS 16, selects theories, and combines cases to evaluate the risks of overseas investment that are difficult to quantify, and analyzes the risks of overseas investment by *Z*-value models and market effects. This research method has pertinence and applicability to enterprises investing overseas.

## Introduction

Since the 21st century, China’s economic policies have continuously guided and supported the methods and directions of overseas investment, and more and more enterprises have participated in the process of overseas investment (Hussain et al., 2020; Kang et al., 2021). It can be said that China’s overseas investment has now achieved fruitful results. Overseas investment has become the only way for enterprises to become bigger and stronger and achieve internationalization. In recent years, the development momentum of overseas investment by Chinese enterprises has been particularly rapid (Xu et al., 2021). By 2021, China’s overseas investment will increase by about 50% year-on-year, accounting for about 20% of the world’s total. This shows those in recent years, national policies, especially the “One Belt, One Roa” guidelines, have indeed provided an important impetus to the development of China’s overseas investment (Lee, 2021; Liang et al., 2021; Wang et al., 2021). However, with the policy guidance of International Financial Reporting Standards (IFRS), overseas investment has been greatly affected. IFRS can promote the healthy development of overseas investment and effectively prevent the results of various risks, which play a key role in strengthening the safety and prudence of overseas investment in the capital market (Xu, 2020).

With the development of economic globalization to this day, it is difficult for companies that are “closed to the country” to survive in the market. To develop, we must face the challenges and risks of overseas investment. There are many potential risks of overseas investment, some are unpredictable, and some can be warned in advance and take countermeasures (Hudakova et al., 2018; Vychytilova et al., 2020). What we need to do is to turn the unpredictable into the predictable, transform the random risk into a controllable risk, and make the loss caused by the risk from large to small (Paskaleva and Stoykova, 2021). In this process, we must first recognize and evaluate risks before we can do a good job of early warning and prevention or reduce risks, reduce uncontrollable obstacles to overseas investment of enterprises, and inject vitality into the stable development of enterprises.

Home appliance manufacturing is an indispensable and important part in the history of industrial development in all countries in the world. As early as the 1990s, China’s home appliance manufacturing industry opened the door to the world with its durable quality and affordable products. *Haier* is China’s oldest home appliance manufacturing company and a pioneer in the “going global” of home appliance manufacturing. During the promotion of the “One Belt, One Road” initiative, *Haier* followed the pace of the policy and went global step by step. At present, *Haier*’s internationalization strategy, global brand strategy and Internet development strategy have important influence on Chinese overseas investment enterprises. Because overseas business accounts for more than half of *Haier*, and its overseas investment has a history of more than 10 years, *Haier* is sufficiently representative to become a case enterprise of overseas investment risk in this paper.

The current overseas investment of Chinese enterprises is an era of coexistence of opportunities and risks. The overseas investment of enterprises has gradually advanced from the requirement of quantity to the level of “winning by quality” (Chen, 2019; Belas et al., 2021). In the context of IFRS, how to increase the quality of overseas investment and reduce the risk of uncertainty caused by factors such as social laws? How to reasonably plan the overseas investment path under the guidance of IFRS? How to overcome the many risk factors of overseas investment and reduce investment failure? This paper takes *Haier*, a benchmark enterprise for overseas investment in China’s manufacturing industry, as an example, and summarizes *Haier*’s investment policies and models from 2008 to 2020. Based on the successful or failed overseas investment cases, the overseas investment risks and risk factors are explored, and some suggestions and inspirations are put forward.

The Section “Introduction” describes the research background and research significance of the paper. The Section “International Financial Reporting Standards (IFRS)” involved in the overseas investment of large companies. The Section “Haier’s Overseas Investment Risk Identification” analyzes the main risks of *Haier*’s overseas investment, and summarizes the characteristics of *Haier*’s overseas investment. The Section “Risk Assessment of Overseas Investment by Haier” is the risk identification, risk assessment, and market reaction of *Haier*’s overseas investment. Among them, risk identification is classified from two levels of macro and business management; risk assessment corresponds to macro and micro levels through qualitative and quantitative research methods; finally, it analyzes the market response to overseas mergers and acquisitions. The Section “Discussion” summarizes the precautionary enlightenment when large companies invest overseas. The Section “Conclusion” summarizes the research work and main conclusions of the full paper.

## IFRS for Overseas Investment

### International Financial Reporting Standards No.9: Financial Instruments

In order to solve the unavoidable drawbacks of International Accounting Standards No.39 (IAS39) in the classification and impairment of financial instruments and achieve accurate classification of financial instruments, International Financial Reporting Standards No.9: Financial Instruments (IFRS 9) came into being. Hopper et al. found that the logical basis of management’s classification of financial assets under IAS39 is often its subjective willingness to hold these financial assets, so the reliability and objectivity of financial statements will be adversely affected to a large extent. Hashim et al. (2016) and Schutte et al. (2020) found that the management’s classification of financial assets under IAS 39 is often motivated by earnings management. When the stock price increases, dividing the financial assets measured at fair value into trading financial assets can increase profits.

The implementation of IFRS has a significant positive impact on enterprises, mainly in improving the quality of accounting information. Joubert et al. (2021) pointed out that the principle of classification of financial assets under IFRS 9 is the business model and contractual cash flow characteristics of the enterprise’s management assets, and he believes that IFRS 9 under this classification principle significantly improves the comparability of corporate financial reporting information and reduces the subjectivity of financial asset classification. Ollaku et al. (2021) pointed out that the implementation of IFRS 9 may have an adverse impact on my country’s financial stability. Al-Sakini et al. (2021) believes that the impact of IFRS 9 on the corporate industry is disruptive, which requires more cost than ever to deal with changes in management models and valuation techniques. Oussama and Jamil (2020) believes that IFRS 9 will increase the difficulty of obtaining audit evidence and increase the cost of auditing, and should allow sufficient preparation time for the implementation of IFRS 9. In addition, the “expected loss model” is inconsistent with the conceptual framework of the current standard system, especially when the enterprise does not have the external market environment and internal management level to implement the expected loss model, which will make the actual operation of the model more difficult. Landini et al. (2019) believes that IFRS 9 is difficult for some enterprises and financial personnel to understand, and it is difficult to fully accept and proficiently apply it in a short period of time. Konstandatos (2020) believes that companies should reasonably assess the possible impact of IFRS 9 and the difficulties encountered.

### International Financial Reporting Standards No.15: Revenue From Contracts With Customers

In May 2014, the International Accounting Standards Board (IASB) and Financial Accounting Standards Board (FASB) issued a new joint standard on revenue recognition (IFRS 15). IFRS 15 states that revenue should be recognized based on the completion of performance of obligations, which is very different from the transfer of risks and rewards as revenue recognition criteria (Khersiat, 2021). Dalkilic argues that revenue is critical to users of financial statements when evaluating an entity’s financial performance and position (Dalkilic, 2014). Aurora and Bontas (2014) noted that IFRS 15 is expected to affect all entities across all industries, but the magnitude of the impact may vary widely. Yeaton proposed that the goal of IFRS 15 is to provide consistent principles for revenue recognition regardless of industry or geographic area. It simplifies current revenue guidance and removes industry- and transaction-specific guidance from existing accounting standards (Arham et al., 2020). Vardiashvili and Maisuradze (2017) pointed out that the introduction of common standards for different contracts in IFRS15 will avoid all the misunderstandings caused by previous standards and provide a more detailed and unified mechanism for accounting.

The FASB and IASB’s joint revenue recognition program has undergone a gradual and slow evolution. For many entities, the number of revenue-related disclosures can increase substantially (Tutino et al., 2019). Jong (2017) analyzed the comprehensive impact of IFRS 15 on the real estate investment strategies of listed hotel companies. Bauer and Centorrino (2017) argue that high-quality financial information is useful to investors and other users of financial statements, and is even more important for entities such as banks that are important to the stability of the financial system. Dani et al. (2017) discussed several changes in IFRS 15 that have largely affected construction companies, promoting the internationalization of customer contract revenue accounting information. Trabelsi (2018) pointed out that revenue recognition under IFRS 15 is carried out on a measured amount basis. In practice, the timing of revenue and profit recognition, capitalization, or contract costs of expenses may be significantly affected, thus leading to significant changes in the measurement of revenue and profit.

### International Financial Reporting Standards No.16: Leases

In 2016, the IASB officially announced to the world that it will fully implement the new lease accounting standard—International Financial Reporting Standards No.16: Leases (IFRS16) in 2019. IFRS 16 introduced the “right of use theory” for the first time, and proposed the accounting treatment of two groups of lessees, which has caused great changes in its initial recognition and subsequent measurement (Lyon, 2010; Cumming and Galt, 2021). As the machines and other related equipment purchased by home appliance manufacturers generally have the characteristics of high rental costs and long lease periods, the relevant changes in IFRS16 will undoubtedly have a significant impact on home appliance manufacturers (Plotnikov and Plotnikova, 2020). The impact of the application of IFRS 16 on the accounting treatment and financial performance of Haier Group is inevitable.

The most obvious changes brought about by the release and implementation of IFRS 16 are the capital structure and current profit and loss of home appliance manufacturers (Beattie et al., 2006). Not only that, the new standards will also play a crucial role in financing, exchange rate risk, and so on. This is a big challenge for home appliance manufacturers. IFRS 16 for home appliance manufacturers, the most obvious change is that the cost of enterprises will be greatly increased, such as accounting costs and financing costs. IFRS 16 is beneficial to the lessee’s leasing business, and it also makes the lessee’s financial status and operating results more authentic (Kliestik et al., 2020; Saad et al., 2020; Lazaroiu et al., 2021). However, in terms of accounting treatment between the lessor and the lessee, the impact will be to change the treatment method. IFRS 16 promotes the development of the leasing industry to a healthy and transparent track, which better reflects the economic essence of the leasing business and requires the cooperation of relevant systems and strategies of enterprises. Under IAS17, the financial report of an enterprise cannot accurately reflect the true solvency and growth ability, which hides some of the financial risks of the enterprise. IFRS 16 effectively makes up for the loopholes in IAS17. Under the influence of this model, accounting becomes more fair and the problem of off-balance sheet financing is solved to a certain extent.

## Haier’s Overseas Investment Risk Identification

### Political Risk

#### Fluctuations in Diplomatic Relations Affect the Political Environment

The development direction of a country’s foreign policy is an important factor influencing overseas investment. For example, due to the tense relations between China and the Trump administration in 2017, the United States issued a series of trade protection policies and patent policies to sanction China, making it difficult for *Haier*’s products to enter the United States. In 2016, China had tense relations with Southeast Asian countries such as the Philippines because of the South China Sea issue, which was also one of the factors that made overseas investment assets at risk of loss. On the contrary, the political environment of countries with good relations with China will have certain advantages. For example, South Africa and other BRICS countries not only provide convenient locations for *Haier* branches, but also introduce a series of policies to protect Chinese enterprises, ensuring the smooth development of *Haier*’s processing bases in South Africa.

#### Risk of Party Disputes, National Sovereignty, and Territorial Disputes

From the perspective of diplomatic relations, China has established diplomatic relations with Southeast Asian, South Asian, and Latin American countries very early. Pakistan and Venezuela, in particular, have close ties to China. *Haier* followed national policy with its own overseas investments. However, Pakistan, Thailand, Iran, Venezuela, and other regions have high political risk ratings, and the political situation at home and abroad is extremely unstable, such as street demonstrations, violent conflicts, and partisan struggles. As a result, *Haier*’s overseas bases in these regions have ceased production, and the infrastructure is nearly deserted. In such a political environment, it is naturally very difficult for overseas investment companies to carry out normal production, which will bring losses to *Haier*.

### Economic Risk

*Haier*’s products mainly focus on daily necessities of home appliances. In recent years, in order to meet the high-tech needs of the market, smart mobile products such as mobile phones have also been gradually launched. According to the characteristics of *Haier*’s products, it can be divided into daily necessities and some high-end consumer goods, and the purchase of products requires a certain economic basis as support. This paper believes that the economic risks faced by countries and regions with good economy will be lower, and the economic risks of regions with poor economy will be higher due to slower product purchase speed.

*Haier* has its unique industry characteristics in the home appliance manufacturing industry, such as market saturation rate and market competition. In Western Europe and parts of the United States, overseas sales were less optimistic than expected due to strong and established rivals such as *Siemens*. Similarly, in the East Asian market, *Haier* faces formidable competitors, such as *Panasonic*, *Sony*, and *LG*. At this stage, major brands have started globalization strategies, and fierce market competition is an economic risk factor that has to be considered.

### Legal Risks

With the pace of globalization, *Haier* has now established its own production bases in about 20 countries around the world, and its business scope has expanded to more than 50 countries. At the same time, we cannot ignore a reality: China’s safeguard measures for overseas investment by local companies are very imperfect. As China advocates a free and open international image, the government seldom protects local companies through trade barriers and other means, and laws on overseas investment only restrict foreign companies from doing business in China. Compared with the mature legal systems and overseas investment insurance in Europe and the United States, China still has a long way to go in terms of legal protection of overseas investment to be in line with international standards.

On the other hand, *Haier* had to face another test: legal issues in the host country. India’s legal system, for example, clearly favors local businesses. During the process of *Haier*’s investment projects in India, there have been many disputes that are not conducive to the local development of *Haier*’s enterprises. For example, when Vietnam established overseas factories, due to the imperfect legal supporting facilities, it increased the approval time for *Haier*’s landing, and tedious procedures. In the United States and some European countries, *Haier* faces more intellectual property risks. In recent years, *Haier* has gradually moved closer to the high-tech industry and occupied the American market in the high-tech field. In the process of overseas investment, only by thoroughly studying the laws and policies of the host country can the impact of legal risks on enterprises be reduced to a certain extent.

### Cultural Risk

When *Haier* acquired *GE* in 2016, the two parties had a series of running-in processes in the process of integration. *GE* prefers young employees with creativity and passion; *Haier* prefers mature employees with experience and qualifications. The conflict between the two in managing enterprises reflects the difference between Chinese and Western cultures. In the end, *Haier* retained most of *GE*’s talent and corporate construction structure, and retained its corporate culture, but the cultural conflict between the two sides in terms of management methods could not be completely eliminated.

*Haier* has also experienced religious and cultural barriers to overseas investment. The differences between *Haier* and the Islamic culture and customs of Saudi Arabia, the United Arab Emirates, and other places have made residents have cultural barriers and resistance to *Haier*, which is not conducive to companies recruiting employees and selling products locally. In Europe and the United States and other countries, with the improvement of the education level of local residents and the high level of people’s living standards, the possibility of cultural risks will gradually decrease. Generally speaking, cultural risk is inversely related to the quality of local residents, and the degree of religious belief will also have a certain relationship with the evaluation of *Haier*’s overseas investment returns.

### Epidemic Risk

Taking the Japanese market as an example, as early as the early stage of the epidemic, *Haier* actively prepared a “breakout plan,” made a double guarantee for production and sales, turned “crisis” into “opportunity,” and achieved a contrarian growth in sales performance. Specifically, the domestic epidemic spread rapidly at the end of January, and *Haier* relied on the global supply chain system to ensure the stable supply of its products in the Japanese market from February to March, and there was no shortage of stock. At that time, when many brands were having headaches due to the impact of the epidemic on their export business, *Haier* was not affected because of its adherence to the “trinity” layout. GfK data show that from January to February 2020, *Haier*’s new life small refrigerator sales increased by 13% year-on-year, and freezers increased by 25% year-on-year; in March, *Haier*’s refrigerator share reached 15.7%, ranking first in the Japanese market.

On the whole, under the overall decline of the market during the epidemic, *Haier* achieved good sales and became the only brand in the Japanese home appliance market to grow against the trend. Behind this set of excellent data, *Haier*’s perfect plan is inseparable. For example, in the early days of the epidemic, many local companies and brands in Japan suspended their new product launch plans and negotiation plans, but *Haier* responded according to a sound plan, insisted on introducing new products, and continued to interact with channels through cloud means.

### Cost Risk

Most of the company’s business still comes from the production of traditional household appliances. Through the analysis of the cost structure of *Haier*, as shown in Table 1, 86% of the production cost comes from raw materials, and more than 6% comes from labor costs. Therefore, price fluctuations of raw materials and labor costs are the main components of *Haier*’s costs.

**Table 1:** Cost analysis of Haier.

Year	Cost item	
	Raw materials	Direct labor
2019	7,625,896.27	614,318.47
Share of total cost %	85.97	6.93
2018	5,450,795.44	406,834.63
Share of total cost %	85.92	6.41
Changes in the same period	39.90	51.10

Data source: Haier group annual report compilation. Unit: 10,000 yuan.

Before the COVID-19, *Haier*’s labor cost in 2019 was 6.1 billion yuan, which was an increase of about 2 billion yuan compared to 2018. This is inseparable from the rise in labor production costs in Southeast Asia. Most of *Haier*’s overseas processing plants are located in economically backward Southeast Asia. Although the *per capita* daily wage of workers is low, with the economic development of Southeast Asia, the problem of rapid labor force growth in some countries has gradually emerged.

“Forbes” published an article in October 2019 saying that workers in Malaysia, Thailand, and Myanmar require wage increases of more than 50% in 2020. As shown in Table 2, Cambodian Prime Minister Hun Sen raised the minimum wage standard for manufacturing workers by 11%. According to Reuter’s statistics, wages for Cambodian processing and manufacturing workers have risen by 150 percent in the past 5 years. Thailand’s labor ministry raised the minimum wage by 3% in October 2019, and plans to continue raising it to about $550 a month. Myanmar officials raised workers’ monthly wages from $82 to $105, an increase of 28 percent. Malaysia has raised its minimum wage by 11% in the past 3 years. Labor costs in Southeast Asia have been rising in the past few years. For the labor-intensive *Haier*, labor cost risks exist.

**Table 2:** Changes of minimum monthly salary standards in some Southeast Asian countries.

Nation	2020 salary standard (monthly salary)	Gain
Cambodia	170	11%
Thailand	224.02	3%
Myanmar	105	28%
Malaysia	237	11%

Data source: Sina finance data. Unit: Dollar.

The cost of raw materials is a key indicator that determines the increase or decrease of its cost. *Haier*’s cost structure and production model determine that its costs will be affected by fluctuations in domestic and foreign metal raw material prices. In particular, the prices of overseas raw materials are difficult to predict and control, which greatly increases the possibility of cost risks. Compared with changes in domestic raw material prices, *Haier* faces a greater challenge in cost risk from foreign raw material markets. As shown in Table 3, taking the Sino-US trade war friction in 2018 as an example, Trump imposed tariffs on imported steel and aluminum, which greatly increased the cost of United States steel mills. This trade protection policy has also led to a surge in the price of raw materials in the United States market, and the rise in the price of production necessities is bound to greatly increase *Haier*’s production costs in the United States.

**Table 3:** North American market steel price list in March.

	Time	Hot roll	Cold plate	Plate	Rebar
North American steel mills	Beginning of the month	789	970	826	694
	End of month	943	1,102	1,003	760
	Increase %	19.52	13.61	21.43	9.51

Data source: data collation. Unit: USD/ton.

As can be seen from the above, due to the start of the trade war, the price of steel raw materials in the United States rose by 10%–20% in March alone. As the Trump administration’s trade protectionism intensifies, *Haier*’s subsequent production orders will face the problem of sharply rising costs, and *Haier*’s development has also been adversely affected by the Sino-US trade war. To sum up, this sudden cost increase is difficult to predict in the process of overseas investment, and the unpredictability of cost changes is enough to threaten the income and profits of enterprises.

### Operational Risks

This paper intercepts the changes in *Haier*’s financial data from 2011 to 2021 as a sample, and focuses on the changes in *Haier*’s performance from the start to the completion of overseas investment projects.

#### Decreased Solvency of the Enterprise

In 2016, *Haier* acquired *GE* for $5.58 billion. According to the agreement between the two parties, 40% of *Haier*’s own assets and 60% of loans obtained through financing. *Haier* said that this part of the loan will be repaid through the income of the subsidiary, and promised that the asset integration will not lead to *Haier*’s asset-liability ratio exceeding 70%. This article sorts out the changes in *Haier*’s solvency indicators from 2011 to 2020, as shown in Table 4.

**Table 4:** Debt solvency of Haier in some years.

Reporting period	Assets and liabilities	Current ratio	Quick ratio
2011/12/31	70.95%	1.21	0.98
2012/12/31	68.95%	1.27	1.04
2013/12/31	67.23%	1.7	1.12
2014/12/31	61.18%	1.43	1.25
2015/12/31	57.34%	1.18	1.16
2016/12/31	71.31%	0.95	0.74
2017/12/31	69.13%	1.15	0.87
2018/12/31	67.58%	1.26	1.07
2020/12/31	62.11%	1.33	1.16
2021/12/31	49.98%	1.48	1.28

As shown in the table, *Haier*’s asset-liability ratio in 2012 and 2016 reached more than 70%, which has violated *Haier*’s announcement when it acquired *GE*, which further shows that the development of the company’s asset-liability ratio has exceeded the company’s expectations. For a large-scale manufacturing enterprise such as *Haier*, such a high asset-liability ratio will inevitably lead to high corporate operational risks. *Haier*’s current ratio fell below 1 for the first time in 2016, due to the huge cash outlay when it acquired *GE*. It can be seen that there were some crises and risks in the capital situation of *Haier* in 2016, and these risks were caused by the failure of the subsidiary to complete the profit plan according to the predetermined target, which increased the repayment cost of corporate loans. At the same time, due to the impact of the COVID-19 in 2019–2020, *Haier*’s overseas investment situation is not optimistic. What is more serious is that due to changes in the company’s financial indicators, *Haier* has been downgraded in the ratings of many credit rating agencies in 2017, and the difficulty of financing for further development of the company has been greatly tested.

#### Increase of Various Expenses

*Haier*’s various expenditures have undergone major changes with the company’s overseas investment and mergers and acquisitions. As shown in Table 4, taking 2015–2017 as an example, after the acquisition of *GE*, *Haier*’s cost of long-term borrowing and financial expenses increased by 2 billion yuan. According to the annual report, *Haier* also incurred a series of exchange losses during the merger and acquisition process, which is another reason for the sudden increase in financial expenses. The net cash flow from investing activities showed a negative growth, and the amount was huge, indicating that *Haier* has been in a stage where the investment outflow is far greater than the inflow in recent years. The recovery period of investment funds will also affect the quality of the company’s cash flow, and long-term negative values can easily put a lot of pressure on the company’s capital chain (Table 5).

**Table 5:** Changes in some financial statement items of Haier.

Subject	2015-12-31	2016-12-31	2017-12-31
Long term loan	2.972	155.3	160.4
Financial expenses	−5.139	7.204	13.93
Non-operating expenses	0.9592	3.362	2.616
Net cash flow from investing activities	−14.49	−28.49	−113.5
Asset impairment loss	3.063	4.905	6.559

Data source: wind data collation. Unit: 100 million yuan.

#### Instability of Corporate Profitability

This paper intercepted the net profit margin, net profit rate of the total assets and Return on Equity (ROE) of *Haier* since 2011. This paper longitudinally analyzed the changes in corporate profitability before and after overseas investment, and introduced relevant indicators of companies in the industry such as *Midea* and *Gree* to horizontally compare the profitability of *Haier*. The results are shown in Table 6.

**Table 6:** Haier profitability index.

Reporting period	Net profit margin	Net profit rate of the total assets	ROE
2017/12/31	5.68	6.4	23.59
2016/12/31	5.62	6.46	20.41
2015/12/31	6.6	7.85	16.22
2014/12/31	7.54	9.84	27.58
2013/12/31	6.42	10.03	32.84
2012/12/31	5.46	9.75	33.78
2011/12/31	4.95	10.57	31.33

Data source: Haier annual report compilation. Unit: percentage.

**Figure 1:**
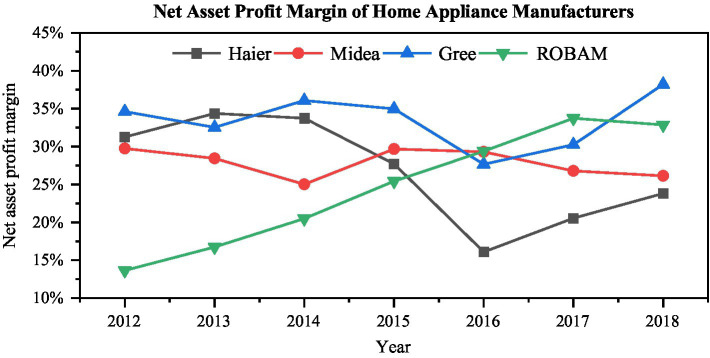
Comparison of profitability between Haier and similar companies.

As shown in Figure 1, since *Haier* acquired Japan’s *Sanyo Electric* and Australia’s *Fisher & Paykel* in 2012, *Haier*’s net profit margin had continued to grow. This showed that the overseas acquisition of the subsidiary had achieved a good synergy effect, which had played a positive role in the sales profit of the parent company. ROE, on the other hand, fell slightly as *Haier* borrowed a lot of money for overseas investments, increasing its debt ratios and equity ratios. In general, the scale of overseas investment in 2012 was relatively small, and the synergy effect was good, which brought a very good positive profit growth to the company. After 2015, due to the acquisition of *GE* and the establishment of production bases in Russia and India, *Haier*’s debt increased and its ROE plummeted. At the same time, the larger management model of *GE* and *Haier* made the synergy effect of the company less than ideal, causing the net sales profit rate to plummet.

Before the acquisition of *GE* in 2015, *Haier*’s profitability in the home appliance industry had been leading the industry. After experiencing large-scale overseas investment, *Haier*’s net profit margin had fallen sharply. On the one hand, it was the borrowing of a large number of loans; on the other hand, it was the problem of the synergy between the subsidiary and the parent company. Therefore, it can be seen that overseas investment is a test for the profitability of enterprises, and there are specific performance risks.

#### The Increase in the Risk of Bad Debts of Enterprises

In 2017, *Haier* continued to make additional overseas investments and added three new accounts receivable as shown in Table 7. Since the above three companies are all located overseas, it is difficult to track the development of the companies in real time, so it is impossible to correctly judge the solvency of the debtor. As a result, it is difficult to predict the future recovery of receivables, and the operating uncertainty is also greatly increased. *Haier* not only had to lose the time value of funds, but also bear the risk of bad debts.

**Table 7:** Haier’s new accounts receivable in 2017.

Client’s name	Accounts receivable	
	Book balance	Bad debt provision
HAIER International Co., Ltd.	43,154,437	2,157,721
HNR Company, Ltd.	122,775,678	6,138,783
Fisher and Paykel Appliances, Ltd.	317,751,221	15,887,561

Data source: Haier annual report. Unit: yuan.

### Exchange Rate Risk

The impact of exchange rate risk on *Haier*’s overseas investments is mainly reflected in exchange rate changes and interest rate differences. In recent years, with *Haier*’s massive increase in overseas investment business, overseas sales have also exploded. According to the contract rules in the field of home appliances, home appliance companies need to bear the gap between “contract currency” and “cost currency.” Therefore, the loss of currency depreciation will be borne by *Haier* before the order is finally completed. After the collapse of the Bretton Woods system, most of the international currencies adopted floating exchange rates that changed daily, especially from 2016 to 2018, the RMB has been appreciating. For *Haier*, contract returns are unpredictable and exchange rate risks are high.

With the increase of overseas infrastructure construction, *Haier*’s loan volume is also gradually increasing, as shown in Table 8. The investment in infrastructure is different from the short cycle of investment and sale of products. The construction of infrastructure takes several years. Haier’s large domestic loans through export credit will generate more financial expenses, thereby reducing corporate profits.

**Table 8:** Proportion of overseas sales in Haier’s sales revenue from 2015 to 2018.

Year	2015	2016	2017	2018
Total sales	89,996,359,668	119,246,700,635	158,726,338,535	177,183,253,252
Overseas sales	18,699,302,564	47,517,434,312	67,039,657,012	71,220,361,228
Mainland sales	71,297,057,103	71,729,266,323	91,686,681,523	105,442,960,624
Proportion of overseas sales	21%	40%	42%	40%

Data source: Haier annual report. Unit: yuan.

As can be seen from Table 8, from 2016 to 2018 after *Haier* acquired *GE*, the proportion of overseas sales was always around 40%. This shows that *Haier* is increasingly reliant on the proportion of overseas revenue, which also increases the impact of exchange rates on *Haier*’s operating income to a certain extent.

In 2016–2017, *Haier* had a considerable amount of foreign currency liquidity and accounts receivable, as shown in Table 9. From 2016 to 2017, the US dollar, the euro and the Japanese yen all showed exchange rate fluctuations of different magnitudes. Enterprises may take profit risks at any time due to changes in exchange rates when delivering orders. On the basis of the continuous expansion of the foreign investment scale of *Haier*, the scale and quantity of orders are also increasing. Therefore, the exchange rate risk faced by *Haier* in each batch of orders cannot be ignored either.

**Table 9:** Haier’s foreign currency and monetary funds in 2016–2017.

Project	2016 Closing balance			2017 Closing balance		
	Foreign currency	Exchange rate	RMB	Foreign currency	Exchange rate	RMB
Money Funds						
Dollar	387,284	6.937	2,686,593	1,249,816	6.5342	8,166,548
EUR	21,250	7.3068	358,823	20,058	7.8023	156,501
JPY	5,184,441	0.0596	308,946	5,007,949	0.0579	289,875
Accounts Receivable						
Dollar	976,653	6.937	6,775,045	1,036,244	6.5342	6,771,029
EUR	49,108	7.3068	358,823	46,517	7.8023	362,939
JPY	4,314,376	0.0569	257,098	3,875,030	0.0579	224,298

Unit: 1,000 yuan.

## Risk Assessment of Overseas Investment by Haier

### Macro Analysis

#### Political Risk

Due to the continuous friction in the trade war, some European countries have imposed various sanctions on China, and overseas investment is also facing higher political risks; in the country risk rating for overseas investment issued by the Ministry of Commerce, the sharp decline in the United States was also affected by this (Table 10). In addition, the stability of the domestic government of the host country also has a huge impact. There are territorial and sovereignty disputes in South Asia, India, and Pakistan; armed struggles and terrorist attacks have occurred in the Middle East for a long time; Africa and Latin America are also hot spots for political risk.

**Table 10:** Changes in political risk in some of Haier’s overseas investment countries.

Nation	2016 Ranking	2016 Ranking	Change	Nation	2016 Ranking	2016 Ranking	Change
America	7	12	Rise	Japan	8	6	Rise
Germany	5	2	Rise	Iran	48	49	Drop
Italy	19	12	Rise	Saudi Arabia	20	23	Drop
Vietnam	40	37	Rise	New Zealand	3	5	Drop
Thailand	41	24	Rise	Nigeria	54	36	Rise
Pakistan	46	47	Drop	South Africa	17	19	Drop
India	22	22	——	Venezuela	56	51	Rise
South Korea	14	13	Rise				

Data source: China overseas investment country risk rating.

#### Economic Risks

Many industries of *Haier* are located in high economic risk areas. These countries or regions take agriculture as their main economic pillar and cannot compare with developed regions in terms of the perfection of supporting facilities. The economic security of these regions is also a severe test for *Haier*. This conclusion is also consistent with the economic indicators in the overseas investment risk rating of the Chinese Academy of Social Sciences. Therefore, this paper adopts the ranking of economic fundamental risks of different countries in the report (Table 11) as the evaluation index of economic risk.

**Table 11:** Economic risk ranking of overseas investing countries in 2017.

Nation	Ranking	Change from previous year	Nation	Ranking	Change from previous year
America	1	——	Thailand	41	Rise
Japan	4	Rise	Vietnam	42	Drop
new Zealand	5	Drop	Iran	47	Rise
Germany	6	Rise	Nigeria	50	Drop
India	22	Rise	South Africa	51	Drop
Pakistan	34	Drop	Venezuela	57	——

Data source: China overseas investment country risk rating.

#### Epidemic Risk

Faced with the dilemma of declining user visits and fewer users during the COVID-19, *Haier* actively upgraded user experience scenarios and put high-end series of refrigerators in the live exhibition hall for experiential display. Users can experience the unique charm of the product in actual use scenarios.

*Haier*’s grasp of pre-plan planning is also one step ahead. During the epidemic, the *Haier* team began to conduct online training for direct sales staff and added 20 employees. With the easing of the COVID-19, the regular contacts of users have gradually increased. After the state of emergency was lifted in Japan in June, these direct sellers were directly introduced into the store. According to the relevant person in charge of *Haier*, the *Haier* team will continue to increase the training of direct sales personnel, and the direct sales personnel will expand the team to several hundred.

#### Cultural Risk

*Haier* had delayed construction in Qatar due to ignoring religious differences; after the acquisition of *GE* in the United States, there was a conflict between *Haier* and *GE* due to the integration of management methods; workers have gone on strike in places such as Venezuela and South Africa because *Haier* employees are unfamiliar with the local language. Through sorting, the author finds that cultural risk has obvious correlation with geographical area. Culture and religion in South Asia, East Asia, and other places rarely cause economic losses; due to differences in management methods, there are often frictions in developed regions in Europe and the United States; due to differences in religious beliefs and daily routines, West Asia, Africa, and Latin America often cause considerable economic losses in terms of manpower.

This paper evaluates the overseas investment risk of *Haier* in different countries from five levels: very high (ranked above 40, there is a big difference with domestic laws, and serious cultural conflicts have occurred), high (ranked 30–40, imperfect laws, and cultural differences), medium (ranked 20–30, the laws are relatively complete, and the cultural differences are small), low (ranked 10–20, the legal system is perfect, and the culture is compatible), and very low (ranked in the top 10, with sound laws and high cultural tolerance), as shown in Table 12.

**Table 12:** Country risk assessment of Haier’s overseas investments.

Nation	America	Germany	Pakistan	Vietnam	Thailand	Japan	Iran	South Africa	Venezuela
Political risk	low	very low	very high	high	medium	very low	very high	low	very high
Economic risk	very low	very low	high	very high	very high	very low	very high	very high	very high
Legal risk	medium	very low	high	high	high	very low	high	high	very high
Cultural risk	very low	very low	high	high	high	very low	very high	high	very high

Data source: China overseas investment country risk rating.

This paper analyzes the overseas investment environment of *Haier* from four macro perspectives. The developed Western European economies represented by Germany have very low risks when accepting overseas investment from *Haier*; East Asian neighbors such as Japan and South Korea have a natural low-risk advantage when accepting *Haier*’s investment. As a developed country, the United States has a high political and legal risk, which is inextricably linked to trade protectionism and the trade war with China to a certain extent. South Asia represented by India and Pakistan and Southeast Asian countries represented by Vietnam and Thailand are affected by the level of economic development and have higher investment risks. In the Middle East region represented by Saudi Arabia, due to its turbulent situation, United States economic sanctions and local Islamic culture, the integration of overseas investment is low, and the investment effect is mediocre. The BRICS countries represented by South Africa have a friendly relationship with China politically, which reduces investment risks. Although the third world countries represented by Venezuela and Nigeria have good political relations with China, it is difficult to eliminate the huge risks brought by domestic and foreign turmoil.

### Enterprise Performance Analysis

In this paper, *Z*-value model is introduced to comprehensively deal with multiple financial variables to obtain the risk of enterprise operation. In the calculation process of the model, five variables and 10 corresponding financial indicators are weighted and averaged, and the risk level of enterprise operation is judged according to the size range of the *Z* value of the conclusion. According to Altman’s point of view, the smaller the *Z* value, the greater the financial risk of the enterprise, and the higher the possibility of facing bankruptcy; and the larger the *Z* value, the safer the enterprise, and the better the operating ability.

The calculation formula of this model is as follows:


$$Z=1.2X_{1}+1.4X_{2}+3.3X_{3}+0.6X_{4}+0.999X_{5}$$

In the formula,

*X*_1_ = (current assets-current liabilities)/total assets;

*X*_2_ = retained earnings/total assets;

*X*_3_ = EBIT/Total assets;

*X*_4_ = book value of shareholders’ equity/total liabilities; and

*X*_5_ = operating income/total assets.

In the model, *X*_1_ is related to the company’s working capital and reflects the company’s solvency; *X*_2_ is the summary of the company’s operating years and operating conditions in the past years; *X*_3_ is the performance of the company’s operating ability without considering tax; *X*_4_ is the ratio of the company’s owner’s equity to debt, which measures the company’s ability to make profits for shareholders and the asset-to-debt ratio; and *X*_5_ is an intuitive reflection of the company’s sales level and profitability.

According to Professor Altman, the criteria for judging *Z* value and company risk are provided in Table 13.

**Table 13:** Judgment standard of *Z* value and company risk.

*Z* score	Probability of bankruptcy in the short term
<1.8	Serious financial crisis and high risk of bankruptcy (high-risk area)
1.8 ~ 2.99	Financial crisis and bankruptcy risk exists (grey area)
>2.99	Little financial crisis and low risk of bankruptcy (low-risk area)

This paper uses the *Z* value of *Haier* in the years before and after the *GE* merger in 2016 to calculate results of *Z* value of Haier.

As can be seen from Table 14, in 2014–2015, *Haier* had good *Z* value data. According to Altman’s statistics, only about 30% of the manufacturing enterprises have a *Z* value range above 2.99, which shows that *Haier* was a manufacturing enterprise with low risk and good operating conditions in 2014–2015. In 2016, subject to the huge borrowing costs for the acquisition of *GE*, *Haier* had a *Z* value lower than 1.8 for the first time. This is in a very dangerous position in the *Z* score model, with a lot of financial risk. In 2017, the *Z* value had a certain degree of correction. Although *Haier*’s operating conditions have improved (*Z* value: 1.6892 → 2.0287), it is still lower than the excellent value before overseas investment (2014: 3.0287, 2015: 2.8903). It can be seen that due to its large long-term borrowings, large investment scale, long capital recovery period, and unstable synergy effect, *Haier*’s operating risks have been in a dangerous state for a long time, and it will take a long time to restore its original foundation.

**Table 14:** Calculation results of Z value of Haier.

Unit: 100 million yuan				
Haier	2014/12	2015/12	2016/12	2017/12
Current assets	594.75	548.67	695.16	820.36
Current liabilities	416.28	397.83	734.53	761.94
Total assets	750.06	759.61	1312.55	1444.49
Total liability	458.86	435.58	936.75	1004.45
Operating income	887.75	897.48	1190.66	1191.9
Retained earnings	148.8	159.32	196.18	237.56
Profit before tax	80.87	69.75	81.83	80.88
Interest expense	−2.31	−4.98	7.21	8.71
Book value of shareholders’ equity	565.33	607.42	602.45	920.1
*X*_1_	0.2379	0.1986	−0.03	0.0404
*X*_2_	0.1984	0.2097	0.1495	0.1645
*X*_3_	0.1042	0.0853	0.0678	0.0808
*X*_4_	1.232	1.3945	0.6431	0.916
*X*_5_	1.1836	1.1815	0.9071	0.8251
*Z* value	3.0287	2.8903	1.6892	2.0287
*Z* value describes the result	Low risk	Grey area	Very high risk	Grey area

Data source: Haier’s annual report compilation.

### Market Response to Overseas Mergers and Acquisitions

*Haier* has carried out many overseas mergers and acquisitions in the 10 years from 2008 to 2018. This paper selects two events, *Haier*’s merger with Japan’s *Sanyo Electric* on July 29, 2011, and its merger with *GE* on January 15, 2016, to explore the different market reactions and intensities of overseas investment of different scales. Among them, *Haier*’s acquisition of *Sanyo Electric*, which cost US$970 million, is the beginning of *Haier*’s white goods acquisition business; and the acquisition of *GE* is *Haier*’s current largest overseas investment, reaching US$5.4 billion. The process is as follows:

For the estimation of this event model, the market model is used:

$\begin{array}{c} R_{\mathrm{it}}=\alpha_{i}+\beta_{i}R_{mt}+\varepsilon_{\mathrm{it}}\\ E\left(\varepsilon_{\mathrm{it}}\right)=0;VAR\left(\varepsilon_{\mathrm{it}}\right)=\sigma \varepsilon_{\mathrm{it}}^{2} \end{array}$

where *R_it_* is the return of security *i* and the market portfolio in period *t*, *ε_it_* is the random disturbance term, and the variance is $\sigma \varepsilon_{\mathrm{it}}^{2}$ .

The research node of *Haier*’s acquisition of *Sanyo Electric* is:

Event Estimation Window: [−130, −6]

Event Date: 2011.7.29

Event window period: [−5,5]

The estimated market model is used to calculate abnormal returns (Table 15) during the window period, the regression results are as follows:

**Table 15:** Changes in abnormal returns.

	Coef	*SE*	*t* stat	*p* value	Upper limit 95%	Lower limit 95%
Intercept	−0.00027	0.001474	−0.18025	0.857253	−0.00318	0.002652
Market return	1.227025	0.133978	9.158426	1.45E−15	0.961824	1.492226

Market Model: $R_{\mathrm{it}}=-0.00027+1.227025R_{mt}$

**Figure 2:**
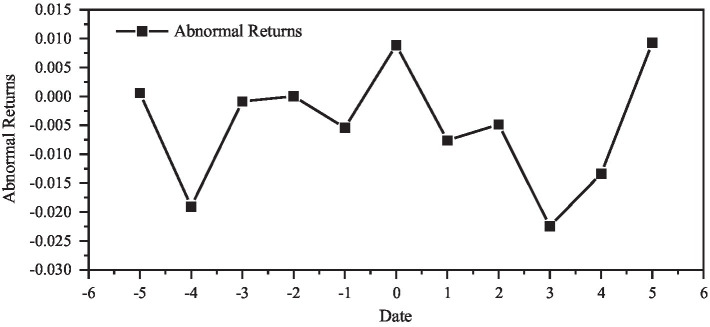
Changes in abnormal returns.

Calculating the Abnormal Return rate (AR) during the event window period, and the result is shown in Figure 2.

It can be seen from the above chart that there is a cumulative abnormal difference rate change before and after the event, but the difference rate is positive or negative, and further testing is needed. The *t* test results are shown in Table 16. It can be seen from the *t* test that the result is not against *H*_0_, and the average abnormal return rate is 0, indicating that *Haier* announced that this merger event did not cause the generation of excess Abnormal Return rate in the market.

**Table 16:** Changes in abnormal returns.

Variable	Obs	Mean	*SE*	*SD*
AR	11	−0.00389	0.002983	0.009892
Mean + mean < AR>	t = 1.3038			
H_0_: mean = 0	Degrees of freedom = 10			
Ha: mean < 0	Ha: mean! = 0		Ha: mean > 0	
Pr(*T* < *t*) = 0.1108	Pr(|*T*| > |*t*|) = 0.2215		Pr(*T* > *t*) = 0.8892	

The research nodes of *Haier*’s acquisition of *GE* are:

Event Estimation Window: [−130, −6]

Event period: 2016.1.15

Event window: [−5,5]

The estimated market model is used to calculate abnormal returns during the window period, the regression results are shown in Table 17:

**Table 17:** Changes in abnormal returns.

	Coef	SE	*t* stat	*p* value	Upper limit 95%	Lower limit 95%
Intercept	−0.00181	0.001195	−1.51913	0.131271	−0.00418	0.00055
Market return	0.943321	0.102269	9.223907	1.07E−15	0.740869	1.145773

Market Model: $R_{\mathrm{it}}=-0.00181+0.943321R_{mt}$

**Figure 3:**
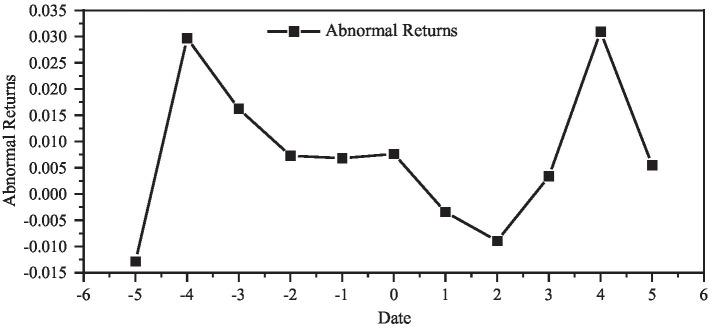
Abnormal returns during the event window.

Calculating the Abnormal Return rate (AR) during the event window period, and the result is shown in Figure 3; Table 18.

**Table 18:** Changes in abnormal returns.

Variable	Obs	Mean	*SE*	*SD*
AR	11	0.007471	0.004205	0.013947
Mean + mean < AR>	t = 1.7767			
H_0_: mean = 0	Degrees of freedom = 10			
Ha: mean < 0	Ha: mean! = 0		Ha: mean > 0	
Pr(T < t) = 0.9470	Pr(|*T*| > |*t*|) = 0.1060		Pr(*T* > *t*) = 0.0530	

The test results significantly support the result that the abnormal return rate is significantly positive, that is, the event produces abnormal excess return rate during the window period.

The author concludes that large overseas investments are more likely to generate excess abnormal returns during the event window because it is easier to leak corporate news or to gain the attention of the capital market. The amount of overseas investment and mergers and acquisitions and the reaction of the capital market basically show the same trend of change. In the decision-making of a relatively large amount of overseas investment, it is necessary to fully estimate the market reaction; otherwise, it is easy to have an impact on the business strategy of the company due to the violent market reaction.

## Discussion

### Actively Responding to National Political and Foreign Policy

In the process of overseas investment, *Haier* always pays attention to the changes in international relations and the foreign policy of the Chinese government, and has established international relations processing departments in overseas branches in the United States, Germany, Japan, and other places. Internally, closely following the pace of the Chinese government’s foreign policy, it has successively established industrial parks in Russia and India. After the BRICS meeting in 2013, *Haier* actively visited India, Brazil, South Africa, and other places to deploy overseas foundries. In the ensuing period, *Haier* has successively established production and R&D bases in South America and Africa, and actively sought help from the Chinese Ministry of Foreign Affairs and the local government. After General Electric of the United States was acquired by Ebanks at an ultra-low price of US$3.3 billion below the market price in 2013, *Haier* actively protested and requested relevant United States departments to strictly investigate the details of the acquisition. In the process of overseas investment, *Haier* attaches great importance to the Chinese government and the host country government, which to a certain extent is conducive to reducing political risks and paving the way for the smooth development of overseas investment.

Therefore, it is necessary to consider the politics and foreign policy of each country when investing overseas. In the system, we should first refer to the national government’s evaluation of the political and economic situation of other countries. When investing in developing countries such as Asia, Africa, and Latin America and in politically turbulent regions, the support of local governments should be actively obtained. When investing in developed countries in Europe and the United States, the other party’s barrier policies and intellectual property protection norms are given priority into consideration, and relevant legal rights protection departments are established to actively use legal weapons to safeguard the legitimate rights and interests of enterprises. Second, the overseas investment system must keep advancing with the changes in the international political situation and the world market. Finally, in order to make decisions scientifically and effectively, it is very important to establish a quantitative evaluation system, such as the EVA system and other performance evaluation and assessment methods, so that the process from prevention to adjustment to feedback is rigorous and scientific, and responds from the root of the risk.

### Strengthening Exchange Rate Monitoring and Making Rational Use of Contract Terms

*Haier*’s prevention of economic risks is mainly reflected in the exchange rate and market. In terms of exchange rate risk prevention, *Haier* has added exchange rate guarantee clauses to its foreign cooperation business since 2015. For foreign business contracts under negotiation, quotations are made based on the expected value of exchange rate changes, and the price is generally calculated based on the expected value of the exchange rate when the revenue is confirmed when the order is delivered. In addition, in some external contracts, the scope of exchange rate fluctuations and the respective responsibilities of both parties are stipulated, and efforts are made to prevent risks in advance. In dealing with risks in the international market, *Haier* has established a financial early warning system to warn risks in advance as much as possible, so as to reduce the harm caused by sudden risks.

### Establishing Legal Departments and Focusing on Legal and Intellectual Property Protection

Since more than 50% of *Haier*’s business is carried out overseas, it has also established dozens of R&D centers overseas. As overseas investments increasingly rely on technology, intellectual property risks are increasing day by day. Since 2015, the legal department of *Haier* has compiled and released the “Legal Analysis Reports on Overseas Investment and Construction” of several overseas investment-related countries every year, striving to protect the cause of overseas investment. Not only that, in order to deal with international intellectual property risks, *Haier* has also established a standard system for independent intellectual property rights. In the past few years, *Haier* has participated in the formulation of more than 10 international standards, more than 200 national standards and hundreds of industry standards related to intellectual property.

After the Sino-US trade war broke out in 2018, *Haier* was accused and sanctioned by the United States government in terms of patents, but the group did not back down and actively approached the Chinese Ministry of Foreign Affairs to gain political support from the Chinese government. At the same time, in the process of going to court with the United States government and companies, they actively collected evidence, mastered US laws, and prosecuted the unreasonable behavior of the United States government, which also showed the United States government’s awareness of Chinese companies’ self-protection. In the end, with the unremitting efforts of the Ministry of Foreign Affairs and the enterprise itself, some malicious lawsuits by the US government and some enterprises were dismissed, and *Haier* took an important step in legal rights protection.

### “Trinity” Localized Management Strategy and Localized Management

In the process of overseas business development, *Haier* actively participates in local economic and social development through localized operation and localized management, and develops together with local communities to prevent overseas cultural risks. *Haier* believes that the overseas development of Chinese home appliance manufacturing enterprises needs to fully integrate into the local culture and take root in the local society. Therefore, in October 2016, through the overseas talent training plan with the school, *Haier* has cultivated many talents with business and engineering combined with foreign language majors, providing a large number of talents for the management of overseas investment companies. In addition, *Haier* pays attention to the integration with local management methods in the investment process. For example, after acquiring Japan’s Sanyo Electric, *Haier* retained all of Sanyo’s staff, and continued to use the Japanese corporate management method unchanged, only making suggestions on the operation of subsidiaries and supervising daily production.

*Haier* recognizes that localization is extremely necessary in cross-border operations. Enterprises should live in harmony with the host country government, win the support of the local government, build a harmonious investment relationship, and quickly integrate into the local social culture in order to become an international enterprise with competitiveness in the local market. Therefore, the way to deal with cultural risks in overseas investment is to respect local religious beliefs and traditional culture to the greatest extent possible. Among the investment projects in Saudi Arabia, in the construction of the base in Saudi Arabia, *Haier* retained the original building, and invested in repairs and reorganization as part of the factory building. Actively integrating into the cultural atmosphere is a point that *Haier* pays particular attention to in the process of overseas investment and construction of infrastructure.

## Conclusion

In order to effectively prevent and solve the challenges and risks of overseas investment, this paper establishes an enterprise risk identification, assessment, and response system, and then analyzes the key projects of *Haier* in the process of overseas investment from 2008 to 2020. It is summarized as follows:

This paper divides the risks of overseas investment into macro risks and micro risks. Macro risks include international political crisis, economic development, market changes, legal risks, cultural integration, epidemic risks, etc., and micro risks include risks such as cost performance of enterprise operations and international exchange rates. Judging from the investment cases of *Haier* over the past 10 years, these risks are basically components of *Haier*’s overseas investment risks.

Therefore, in the risk identification and assessment of *Haier*, this paper introduces *Z* value model and event analysis method as tools, uses qualitative and quantitative aggregation methods, and analyzes the risks of *Haier*’s overseas base construction and investment and merger cases from the perspectives of corporate performance and capital market response. Finally, this paper puts forward reasonable suggestions on the risk prevention mechanism of overseas investment. If we can rationally combine the power of governments and society, and build a reasonable internal system, enterprises can maximize the benefits and risks, and go further and further on the road of overseas investment.

The analysis process of this paper has shortcomings. The risk of overseas investment is the result of the combined influence of multiple factors. When analyzing the causes of some risks, it is difficult for the author to completely remove the interference of other factors (such as climate, etc.), and only analyze some core and important factors. Therefore, research in this area still needs to be strengthened.

## Data Availability Statement

The raw data supporting the conclusions of this article will be made available by the authors, without undue reservation.

## Author Contributions

BZ: writing—review and editing, data curation, and formal analysis. WS: conceptualization and writing—review and editing. TO: investigation, formal analysis, and supervision. HB: resources, visualization, and supervision. CH: resources, formal analysis, visualization, and supervision. All authors contributed to the article and approved the submitted version.

## Conflict of Interest

The authors declare that the research was conducted in the absence of any commercial or financial relationships that could be construed as a potential conflict of interest.

## Publisher’s Note

All claims expressed in this article are solely those of the authors and do not necessarily represent those of their affiliated organizations, or those of the publisher, the editors and the reviewers. Any product that may be evaluated in this article, or claim that may be made by its manufacturer, is not guaranteed or endorsed by the publisher.

## Abbreviations

IFRS: International Financial Reporting Standards
IAS: International Accounting Standards
IASB: International Accounting Standards Board
Haier: China Qingdao Haier Group Co., Ltd
Panasonic: Panasonic Co., Ltd
Sony: Sony Corporation
LG: Lucky Goldstar
Siemens: Siemens AG
GE: General Electric Company
Sanyo Electric: Sanyo Electric Co., Ltd
Midea: Midea Group Co., Ltd
Gree: Zhuhai Gree Electric Appliances Co., Ltd
